# Accuracy of the Hammersmith infant neurological examination for the early detection of neurological changes in infants exposed to Zika virus: A case-cohort study

**DOI:** 10.1097/MD.0000000000029488

**Published:** 2022-06-24

**Authors:** Tathiana Ghisi de Souza, Eduardo Bagne, Renata Mizani, Ali Abdalla Rotob, Rosa Estela Gazeta, Ana Laura de Sene Amâncio Zara, Cohorte Zika virus Jundiaí, Saulo Duarte Passos

**Affiliations:** a Faculdade de Medicina de Jundiaí, Departamento de Pediatria, Jundiaí, SP, Brazil; b Universidade Paulista, UNIP, Jundiaí, SP, Brazil; c Universidade Federal de Goiás, Departamento de Saúde Coletiva, Instituto de Patologia Tropical e Saúde Pública, Goiânia, GO, Brazil.

**Keywords:** arthrogryposis, cerebral palsy, neurological examination, Zika virus

## Abstract

The Hammersmith infant neurological examination (HINE) is a highly predictive tool for the easy and low-cost detection of cerebral palsy. Between 2015 and 2016, the rapid spread of the Zika virus (ZIKV) in Brazil was responsible for an increase in microcephaly cases. This study aimed to verify the accuracy of the HINE for the early detection of neurological problems in Brazilian babies exposed to ZIKV.

This was a cross sectional case-control study of children exposed to ZIKV. This study was part of the Jundiaí ZIKV Cohort. Of a total sample of 782 children, 98 were evaluated (26 in the exposed group and 63 in the control group). We included late preterm infants and term infants who were exposed to the ZIKV and were participants in the ZIKV Cohort study. Student's *t*-test and stepwise multivariate logistic regression were used to compare groups.

Of the 26 items evaluated in the five scored categories of the HINE (cranial nerve function, posture, movements, tone, reflexes, and reactions), only the difference in ankle dorsiflexion between the exposed and the control groups was statistically significant. However, some items showed a significant trend in relation to the control group.

Our results demonstrated the importance of early neurological assessment of infants exposed to ZIKV, even in those without a microcephaly diagnosis.

## 1 Introduction

In 2015, the Brazilian Ministry of Health identified a notable increase in microcephaly cases within the Pernambuco state. An association between the vertical transmission of Zika virus (ZIKV) and fetal abnormalities, such as microcephaly, brain calcifications, agenesis or abnormalities of the corpus callosum, ocular abnormalities, and arthrogryposis, was reported in 2016.^[1,2]^

ZIKV syndrome has highly diverse clinical manifestations of variable intensity. Infection during the first pregnancy trimester usually causes severe fetal abnormalities.^[3,4]^ The gravity of resulting neurological issues requires further elucidation. Although an affected newborn's head circumference may be normal at birth, they may present with cognitive, adaptive, and behavioral changes in the long term.^[5–7]^

In recent decades, the importance of early detection of neurological changes has increasingly been recognized, considering neuroplasticity within the first years of life. Early identification of neurological changes in newborns may help to promote learning and facilitate the planning of appropriate interventions based on specific patient needs, rather than just a general intervention based on the presence of injury. The diagnosis should be made as early as possible using specialized evaluations, such as the General Movements Assessment and Hammersmith's Child Neurological Assessment (HINE), in association with changes observed on magnetic resonance imaging.^[8,9]^

The HINE is a simple instrument for assessing infants aged 2–24 months (chronological or corrected age). It has been proposed as a key tool for the early diagnosis of neurological changes in cerebral palsy (CP). It consists of 37 items in three sections: the first section includes 26 neurological items; the second, 8 items that assess the development of motor functions; and the third, 3 items that evaluate the behavioral state. The evaluation form was translated into Portuguese, was adapted for use in the Brazilian population, and is currently being validated. It provides instructions for carrying out each item's test. It is accessible and simple, requiring no more than 15 min to be performed (see Form, Supplemental Digital Content).^[10–12]^

Recent studies have also demonstrated the association of the HINE protocol in clinical practice to early CP diagnosis and cognitive change detection in children at risk.^[12]^ In underdeveloped countries, where magnetic resonance imaging scans are not widely available, the HINE should be used because it can predict the risk of neurological deficits, such as CP, with 90% to 100% sensitivity and 85% to 100% specificity, and cognitive and sensory changes with 51% to 82% sensitivity and 71% to 90% specificity. A score for asymmetries published recently may also help to predict spastic hemiplegia.^[13–15]^

This study investigated the accuracy of HINE assessment for the early detection of changes in children exposed to ZIKV and described its results to aid multidisciplinary teams regarding possible key changes in exposed children.

## 2 Methods

### 2.1 Participants

This work is an excerpt from the ZIKV∗ Cohort study, which started in March 2016 and ended in December 2021. All data were obtained from a Case-Cohort study in a convenience sample of children aged 3 to 24 months, matched by chronological age or, in cases of preterm newborns, by corrected age, chosen randomly by arrival order, and belonging to the Jundiaí ZIKV Cohort, from March 2017 to February 2020. Further details of the ZIKV Cohort have been described by Gazeta et al and Sanchez et al.^[7,16]^

For this study, infants were selected randomly by order of convenience, at the specialty outpatient clinic of Jundiaí Medical School, which operated on two Saturdays every month from March 2017 to February 2020. All research subjects belonged to the urban cluster of Jundiaí, São Paulo, Brazil (Table 1).

**Table 1:** Sociodemographic characteristics of the mothers and infants participating in the research at the High-Risk Outpatient Clinic of the Jundiaí Medical School, Jundiaí-SP.

Sociodemographic characteristics	Infant
Age (mo) (n = 89)	n (%)
2–6 mo	39 (43)
7–12 mo	24 (26)
13–24 mo	25 (28)
Mother's marital status	Mother
Married	42 (47)
Cohabited	34 (38)
Single	8 (9)
Divorced/separated	2 (2)
Widow	1 (1)
Not informed	2 (2)
Ethnicity	
White	51 (57)
Brown	29 (33)
Black	7 (8)
Indigenous	0 (0)
Not informed	2 (2)
Paid work	
Yes	49 (55)
No	38 (43)
Not informed	2 (2)
Municipality of residence	
Jundiaí	58 (65)
Várzea Paulista	15 (17)
Other municipalities in the microregion	14 (16)
Not informed	2 (2)

Assessed infants were divided into unexposed (control group [CG]) and exposed (exposed group [EG]) groups and were randomized by chronological or corrected age (2–6 months, 7–12 months, and 13–24 months), selected at random in a ratio of 5:2 (CG:EG).

Exposed infants were those whose mothers had at least 1 ZIKV-positive sample by reverse transcription-polymerase chain reaction (RT-PCR) or enzyme-linked immunosorbent assay (ELISA) performed on urine and serum samples during pregnancy or on breast milk samples after birth. Infants with vertical exposure (positive mothers) and a positive RT-PCR test within the first 10 days of life were included. Quantitative real-time PCR (RT-qPCR) was performed using the Creative Biogene Zika Virus Real-Time RT-PCR Kit, as described by Lanciotti et al.^[17]^ All blood samples were also analyzed using ELISA (Euroimmun^TM^, Euroimmun, Lubeck, Germany) to detect IgM and IgG antibodies for dengue, chikungunya, and ZIKV, using their respective Euroimmune commercial kits.^[17]^

HINE evaluations were performed by three trained physiotherapists and the babies were evaluated with the protocol only once; the exposure to zika virus in most cases was only certified after the evaluation due to delay in the exam result, which makes any possibility of bias more difficult. The HINE protocol facilitates early CP diagnosis with 90% sensitivity, when used exclusively, and can predict the level of functional capacity of infants at the age of 2 years using the Gross Motor Function Classification System (GMFCS), starting at 9 months of age. The HINE is a simple, quick, and accessible assessment tool comprising 26 scored items that are divided into five categories, with each test scored from 0 to 3 points. The variables analyzed in this study were the cranial nerves, posture, movements, tone and reflexes, and reactions. The categories of motor milestones and behavior were not scored, although these provided important data about the infant's neuromotor and behavioral development. The necessary criteria for the correct application of the examination are shown in Table 2.

**Table 2:** Criteria used for the correct application of the Hammersmith Infant Neurological Examination.

• The evaluation was carried out between feedings, during the physiotherapy service, in a quiet room, at the multidisciplinary team of the Zika Coorte Jundiaí Project meetings developed by the Jundiaí Medical School.
• Each evaluated item test was performed, and the predominant response was recorded on the evaluation form. When the infant did not fit into any of the possibilities, the closest diagram was chosen.
• All children evaluated were in good clinical condition. For infants who had a strong cry or excessive irritability, the examination was stopped and was resumed at the next meeting.
• In case of prematurity, the assessment was carried out according to the Corrected Gestational Age at the time of the examination.

The total score could be between 0 and 78 points, with optimal values above 73 points, suboptimal at 60–72 points, and altered below 60 points. In younger children, the cutoff points were > 57 for those aged 3 months and > 63 for those aged 6 to 12 months. The lower the score, the greater the neurological changes, and the higher the risk of CP diagnosis. A low score was associated with the highest levels of dependence in the GMFCS classification at 2 years of age. The best prognosis is linked to early identification of the risk of changes in development.^[18,19]^

### 2.2 Ethics approval

The date of birth, gestational age, and data regarding exposure to ZIKV were obtained from the medical records for analysis. Mothers participating in the task force were invited to participate in the project, were given an explanation of the study, and were enrolled once they had given written consent. This work was approved by the Research Committee of the Ministry of Health (*Comitê de Pesquisa do Ministério da Saúde*), under opinion No. 1.446.577.

### 2.3 Sample size calculation

The ideal sample size was calculated to allow detection of an odds ratio of 2.5, considering a study power (1 - ß) of 80%, an alpha error of 5%, and a relative frequency of 10% of a given exposure. This value was utilized as this study analyzed several exposure factors, some of which had an unknown constancy in the original population. The sample size was calculated based on data from 323 infants. The sample size calculation indicated a minimum ratio of 2 cases to 5 controls, to which we added 10% to compensate for possible losses.

### 2.4 Statistical analysis

All data were input into a database prepared using Microsoft Excel (version 2012 Office 365, Microsoft Corp., Redmond, WA). For statistical analysis, the data were stored and analyzed using SPSS V20 (IBM Inc., Armonk, NY), Minitab 16 (Minitab, LLC, State College, PA; https://www.minitab.com/en-us/support/downloads/), and Excel Office 2010 programs (Microsoft Corp.). We compared groups using Fisher exact test for qualitative variables and the Mann–Whitney test for non-parametric numerical variables. Multivariate logistic regression analysis was performed using a stepwise method. For all statistical analyses, a significance level of 5% (*P*-value < .05) was adopted.

## 3 Results

**Figure 1:**
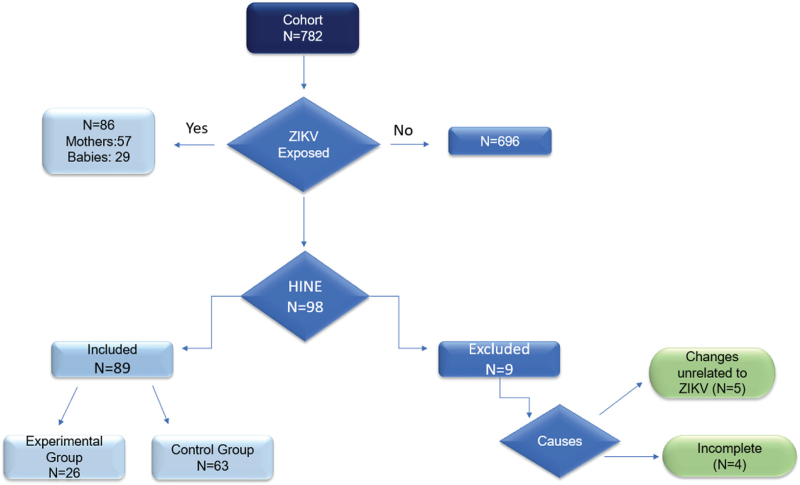
Cohort flowchart for identifying the study participants.

From March 2017 to March 2020, 98 children participating in the Jundiaí ZIKV Cohort were evaluated. Of these, 26 children (mean age: 11.35 months) in the EG and 63 children with an average age of 8.14 months in the CG were included in the study. Nine children (8.6%) were excluded for the following reasons: one infant with Down syndrome, one with congenital cytomegalovirus infection, one with orthopedic deficits (hip dysplasia), two infants aged over 24 months, and four infants with an incomplete evaluation. The study flowchart is shown in Figure 1.

There were more males in the EG (14/53%) and more females in the CG (34/56%). The test used to diagnose ZIKV differed significantly between the groups (*P* < .001); overall, 86 (96.6%) infants were diagnosed by RT-PCR, and three infants were diagnosed by ELISA (3.4%).

In multivariate logistic regression analysis using the stepwise method, only the ankle dorsiflexion test was significant, with a positive coefficient of 1.57. It was thus considered a predictive factor. Children with alterations in the ankle dorsiflexion test were 4.79 times more likely to have been exposed to ZIKV than those who had a typical test response.

Of the 26 evaluated items in the 5 HINE categories, 12 items showed no difference between groups (*P* > .99); ankle dorsiflexion showed a higher average score in the CG than the EG at 2 to 6 months (2.58) and 7 to 12 months (1.75) (*P* = .014). Some items showed a trend for significance, such as the 2 to 6-months hearing response (*P* = .07), positioning of the legs in supine and standing positions (*P* = .09), and hip adductors at 7–12 months (*P* = .09).

When classified into atypical and normal results for each test, Fisher exact test again showed a significant difference between the groups in terms of the ankle dorsiflexion test, but only in infants aged 7 to 12 months, where atypical findings accounted for 37% of the EG and 0.0% of the CG infants (*P* = .024). In qualitative analysis, although there was no significant difference between the groups, the “Posture of the Trunk” item was more frequently abnormal in the EG (62.5%) than in the CG (48.4%). When scores were classified by category, the average score was lower in the EG for cranial nerve function at 7 to 12 months, posture at 7 to 12 months, movements at 7 to 12 months, tonus at 2 to 6 months and 7 to 12 months, and reflexes from to 7 to 12 months and 13 to 24 months. Nevertheless, there was no significant difference in the scores. In the overall general score in the EG, there was only a trend toward significance in the tone category at 7 to 12 months (Table 3).

**Table 3:** Comparison of the items of the Hammersmith Infant Neurological Examination with a trend toward significance.

		EG		CG		Total		
		N	%	N	%	N	%	*P*-value
Auditory response								
2–6 mo	Atypical	0	0%	10	32.30%	10	25.60%	.07
	Normal	8	100.00%	21	67.70%	29	74.40%	
7–12 mo	Atypical	1	12.50%	1	5.90%	2	8.00%	.453
	Normal	7	87.50%	16	94.10%	23	92.00%	
13–24 mo	Atypical	0	0%	1	6.70%	1	4.00%	.6
	Normal	10	100.00%	14	93.30%	24	96.00%	
Legs in sitting, supine, and standing								
2–6 mo	Atypical	5	62.50%	20	64.50%	25	64.10%	.314
	Normal	3	37.50%	11	35.50%	14	35.90%	
7–12 mo	Atypical	4	50.00%	3	17.60%	7	28.00%	.099
	Normal	4	50.00%	14	82.40%	18	72.00%	
13–24 mo	Atypical	0	0%	1	6.70%	1	4.00%	.6
	Normal	10	100.00%	14	93.30%	24	96.00%	
Hip adductors								
2–6 mo	Atypical	1	12.50%	2	6.50%	3	7.70%	.407
	Normal	7	87.50%	29	93.50%	36	92.30%	
7–12 mo	Atypical	2	25.00%	0	0%	2	8.00%	.093
	Normal	6	75.00%	17	100.00%	23	92.00%	
13–24 mo	Atypical	2	20.00%	2	13.30%	4	16.00%	.374
	Normal	8	80.00%	13	86.70%	21	84.00%	
Ankle Dorsiflexion								
2–6 mo	Atypical	3	37.50%	3	9.70%	6	15.40%	.077
	Normal	5	62.50%	28	90.30%	33	84.60%	
7–12 mo	Atypical	3	37.50%	0	0.00%	3	12.00%	.024
	Normal	5	62.50%	17	100.00%	22	88.00%	
13–24 mo	Atypical	0	0%	1	6.70%	1	4.00%	.6
	Normal	10	100.00%	14	93.30%	24	96.00%	

CG = control group, EG = experimental group.

When we classified the global score as optimal, suboptimal, and altered, 80 evaluations yielded an optimal score (>73 points), including 58 from the CG and 22 from the EG, two evaluations with a suboptimal score in the CG and five in the EG, and three altered assessments in the CG and two in the EG. In all altered assessments in both groups, the infants were diagnosed with microcephaly; however, the cases in the CG were related to intrauterine growth restriction rather than to neurological injury. The number of asymmetries per group ranged from 1 to 7, with an average of 4 asymmetries in the EG (two infants) and 1.5 asymmetries in the CG (eight infants) (Table 4).

**Table 4:** Total score and category scores of the Hammersmith Infant Neurological Examination by age subgroup and group.

TOTAL	Average	Median	SD	IQR	N	CI	*P*-value
Cranial nerve function							
2–6 mo							
Exposed	14.00	15.0	2.83	0.0	8	1.96	.304
Control	13.87	15.0	2.05	2.0	31	0.72	
7–12 mo							
Exposed	13.88	15.0	3.18	0.0	8	2.20	.855
Control	14.65	15.0	0.79	0.0	17	0.37	
13–24 mo							
Exposed	15.00	15.0	0.00	0.0	10	- x -	.239
Control	14.27	15.0	2.34	0.0	15	1.19	
Posture							
2–6 mo							
Exposed	13.38	14.0	5.04	2.3	8	3.49	.343
Control	12.65	13.0	3.61	4.5	31	1.27	
7–12 months							
Exposed	15.00	16.0	4.31	3.2	8	2.99	.418
Control	16.53	18.0	1.87	2.0	17	0.89	
13–24 mo							
Exposed	17.80	18.0	0.63	0.0	10	0.39	.486
Control	16.60	18.0	4.37	0.0	15	2.21	
Movements							
2–6 mo							
Exposed	6.00	6.0	0.00	0.0	8	- x -	.366
Control	5.55	6.0	1.46	0.0	31	0.51	
7–12 mo							
Exposed	5.25	6.0	2.12	0.0	8	1.47	.145
Control	6.00	6.0	0.00	0.0	17	- x -	
13–24 mo							
Exposed	6.00	6.0	0.00	0.0	10	- x -	.414
Control	5.67	6.0	1.29	0.0	15	0.65	
Tone							
2–6 mo							
Exposed	20.00	20.0	3.42	6.5	8	2.37	.847
Control	20.16	21.0	3.25	5.0	31	1.14	
7–12 mo							
Exposed	19.13	21.0	6.47	3.7	8	4.48	.067^∗^
Control	22.59	22.0	1.18	2.0	17	0.56	
13–24 mo							
Exposed	23.10	24.0	1.45	1.0	10	0.90	.273
Control	22.37	23.0	1.65	3.0	15	0.84	
Reflexes							
2–6 mo							
Exposed	13.25	14.0	2.19	3.0	8	1.52	.256
Control	11.87	13.0	3.02	4.5	31	1.06	
7–12 mo							
Exposed	13.25	15.0	3.81	1.2	8	2.64	.725
Control	13.82	15.0	2.32	1.0	17	1.11	
13–24 mo							
Exposed	14.90	15.0	0.32	0.0	10	0.20	.460
Control	14.60	15.0	0.91	0.0	15	0.46	
Global Score							
2–6 mo							
Exposed	66.63	70.0	10.29	8.5	8	7.13	.257
Control	64.10	66.0	9.72	11.0	31	3.42	
7–12 mo							
Exposed	66.50	73.0	19.41	5.2	8	13.45	.278
Control	73.59	75.0	3.94	4.0	17	1.87	
13–24 mo							
Exposed	76.80	77.5	1.69	1.7	10	1.05	.216
Control	73.50	76.0	8.93	4.5	15	4.52	

CI = confidence interval, IQR = interval between quartiles, SD = standard deviation.

∗ 7–12 months. Tone.

Thirty-six children aged > 9 months were evaluated (EG, n = 15; CG, n = 21); therefore, a motor prognosis could be made based on GMFCS levels, considering the suboptimal and altered scores. In the EG, only one child had Level III–V motor prognosis (diplegia or severe quadriplegia), and in the CG, one child had level II motor prognosis (hemiplegia or moderate diplegia). The other children, despite having suboptimal scores, were considered typical due to a significant lack of asymmetries and a prognosis of independent walking at 2 years of age. Regarding evaluation accuracy, the values were higher in the 7 to 12-month age group (64%), with the highest specificity of 76% in this group, whereas test sensitivity was highest in the 2 to 6-months age group (50%).

## 4 Discussion

Comparing neurological findings in children with exposure to ZIKV is difficult due to currently limited reports and differing types of methodologies adopted. Most studies that have assessed older children used assessment protocols based on motor development that requires certification and are costly and time-consuming to administer.^[18,20,21]^ The neurological assessment mainly aims to verify the integrity of the central nervous system—the lower the neurological score, the greater the risk of the child being diagnosed with CP.^[13,15]^ Several studies have proposed the use of the HINE to detect central nervous system changes in children exposed to ZIKV; however, these studies have only considered the global score rather than using individual evaluations, which are rich in data when interpreted qualitatively.^[18–22]^ Our findings may have been limited by the small number of children in each group; however, despite most items not showing a significant difference between the exposed and non-EGs, when analyzed qualitatively and separated by categories, this analysis yielded insight because minor changes that may typically not be investigated, but could impact a child's functionality and quality of life in the future. These include the following issues.

### 4.1 Hearing issues

One benefit of the HINE in comparison to other assessment tools is that it not only identifies children at risk for CP but also provides additional information regarding motor impairment type and severity. As this evaluation includes several aspects of neurological functions, it can identify abnormal signs related to other aspects early, such as visual or dietary changes, which is particularly important because CP is not limited to motor impairment.^[23]^ Our findings were similar to those of Aguilar Ticona et al, who found no severe changes in the HINE neurological assessment, but found minor changes in terms of auditory behavior related to orientation, location, and attention to an auditory stimulus except hearing loss.^[18]^ Our findings showed a tendency toward significance at the age of 2 to 6 months in the hearing assessment. Early identification of this change can facilitate the planning of a specific intervention based on function, rather than only on a neurological injury. The multidisciplinary follow-up and monitoring of all children exposed to ZIKV during pregnancy should include early hearing assessment and subsequent follow-up.^[24]^

### 4.2 Changes in posture and tone, particularly ankle dorsiflexion

Some studies have shown a motor delay in 15% of children exposed to ZIKV without microcephaly at birth.^[25]^ In our study of 26 exposed children, only 2 were diagnosed with microcephaly at birth; however, 5 (19%) children had a suboptimal global score, and 2 (7.6%) had an altered global score. The ankle dorsiflexion item was the only test that showed a statistically significant difference between the EG and CGs. The EG had a 4.79 greater risk of atypical findings related to ZIKV exposure. Six EG children showed significant alterations in the examination and had low test scores: one had a positive ZIKV diagnosis after birth, two had a positive ZIKV diagnosis of mother and child after birth, and the other three children were positive for ZIKV diagnosed by maternal examination in the second trimester of pregnancy. These findings are considered important findings because the presence of arthrogryposis is related to ZIKV exposure. Our findings support those of Matos et al (2020), who found changes in 15% of the evaluated babies.^[26]^ The various orthopedic disorders were hypertonia and spasticity, clubfoot, vertical talus, hip dysplasia, knee dislocation, and congenital joint contractures, consistent with congenital multiple arthrogryposis.^[26]^ Arthrogryposis has been reported in fetuses and children with ZIKV infection (11%–85%), as well as clubfoot, congenital hip dislocation, knee flexion contracture, knee hyperextension associated with subluxation, and contractures in hip flexion. Adduction and external rotation have been described as the most prevalent deformities.^[24,25,27]^ The specific mechanism underlying the development of contractures is not clear. The contractions are not always critical, but a simple decrease in the ankle joint range of motion can lead to changes in the crawling stages, pulling up to stand, and walking. Decreased ankle dorsiflexion is strongly associated with ankle injuries in children.^[28,29]^

### 4.3 Leg positioning

The HINE Global Score is sensitive for differentiating children who will walk from those who do not have independent gait. Such a prognosis can be made from 9 months of age, as neurological findings are variable until 9 months but change little thereafter.^[13]^ In our study, only one child in the EG had a global score of 19 and a GMFCS motor level prognosis III–V (diplegia or severe quadriplegia), while one child in the CG had a global score of 42.5 and a level II motor prognosis (hemiplegia or moderate diplegia). A HINE Global Score below 40 points is associated with severe motor impairment.^[30]^ Other children, despite presenting suboptimal scores, were considered typical due to an insufficient number of asymmetries. All children with suboptimal values had an independent gait prognosis, with a global score > 60 points.

### 4.4 Asymmetries

The number of asymmetries is important for differentiating mild impairments from those with a lower score but with typical development. Typically affected children have fewer than three asymmetries in the assessment, while children with mild impairment may have 6 to 15 asymmetries. The presence of four asymmetries should be considered suspicious.^[31]^ In our study, EG children had more asymmetries (average of 4) than those in the CG. Those children need to be monitored by a multidisciplinary team.

### 4.5 Limitations

In our study, the loss of a large number of children may have some implications for the findings and clinical alterations of children exposed to ZIKV; perhaps a greater number of positive children's evaluations could improve the results in relation to the statistical analysis.

In conclusion, our results demonstrate the importance of early neurological assessment in infants with ZIKV exposure, even in those not diagnosed with microcephaly. However, further studies with larger numbers of participants are needed to confirm these results. The HINE protocol is a fast, accessible, and low-cost test that is currently considered highly sensitive for early neurological and cognitive disorder diagnosis. It can be used as a complementary test in, for example, new disease emergence, validation of new neuroprotective treatments, and follow-up programs assessing their impact on the neurological integrity of children under 2 years of age.

## Acknowledgments

We acknowledge all the patients who participated in the study and their families, as well as all the volunteers, researchers, and the interdisciplinary team involved in this project. We acknowledge all members of the research team of the Zika Coorte Jundiaí, specially Alexandra Siqueira Mello, Ali Abdalla Rotob, Alify Bertoldo da Silva, Clovis Antonio Lopes Pinto, Danielle Bruna Lealde Oliveira, Danilla Soares Tam- balo, Diego da Silva Lima, Dirce Takako Fujiwara, Edison Luiz Durigon, Eduardo Roberto Bagne, Fernanda Guerra Velasco, Fernando Novo Arita, Francisco Del Moral Hernandez, Geovane Ribeiro dos Santos, Karen Richter Comandulli, Luiz C P Baran, Márcia Borges Machado, Mayana Zatz, Maria Manoela Duarte Rodrigues, Maria Amélia Farrão, Mirella Nayane Barbosa Leite, Nemésio Flor- ence Vieira Filho, Patrícia Carvalho Loiola, Raquel Prestes, Renata Maria Mizani, Sandra Helena A Bonom, Sergio Vranjac, Sérgio Rosemberg, Tathiana Ghisi de Souza, Viviane Cristina Martori Pandini, and Viviam Paschoarelli Paiva. We would like to thank Editage (www.editage.com) for English language editing.

## Author contributions

**Conceptualization:** Tathiana Ghisi de Souza.

**Data curation:** Ali Abdalla Rotob, Eduardo Bagne, Renata Mizani, Tathiana Ghisi de Souza.

**Formal analysis:** Ana Laura de Sene Amâncio Zara, Tathiana Ghisi de Souza.

**Funding acquisition:** Saulo Duarte Passos.

**Investigation:** Tathiana Ghisi de Souza.

**Methodology:** Tathiana Ghisi de Souza.

**Project administration:** Saulo Duarte Passos.

**Supervision:** Cohorte Zika virus Jundiaí, Eduardo Bagne, Rosa Estela Gazeta, Tathiana Ghisi de Souza.

**Visualization:** Eduardo Bagne, Rosa Estela Gazeta.

**Writing – original draft:** Tathiana Ghisi de Souza.

**Writing – review & editing:** Ana Laura de Sene Amâncio Zara.

## Supplementary Material

Supplemental Digital Content
