# Supplementary material for: Accuracy of the Hammersmith infant neurological examination for the early detection of neurological changes in infants exposed to Zika virus: A case-cohort study
Source: Medicine (Baltimore). 2022 Jun 24;101(25):e29488. doi: 10.1097/MD.0000000000029488 (PMC9276139; doi:10.1097/MD.0000000000029488)
Supplement: Supplemental Digital Content [file medi-101-e29488-s001.pdf]

EXAME NEUROLÓGICO INFANTIL DE HAMMERSMITH (v 19.03.20)

Nome: **Data do nascimento:**

Idade gestacional: **Data do exame:**

Idade cronológica / Idade corrigida: **Perímetro cefálico:**

RESUMO DO EXAME
**Escore Global (pontuação máxima 78)**
**Número de assimetrias**
**Escore comportamental (não faz parte do escore global)**

**Função dos nervos cranianos:**	**Escore**	**(máximo 15)**
**Postura:**	**Escore**	**(máximo 18)**
**Movimentos:**	**Escore**	**(máximo 06)**
**Tônus:**	**Escore**	**(máximo 24)**
**Reflexos e reações:**	**Escore**	**(máximo 15)**
**COMENTÁRIOS**		

(Durante o exame, se uma resposta não for ótima, mas não for ruim o suficiente para marcar escore 1, dê um escore 2)

SEÇÃO 1:EXAME NEUROLÓGICO

AVALIAÇÃO DA FUNÇÃO DO NERVO CRANIANO

	Escore 3	Escore 2	Escore 1	Escore 0	Esc.	Assimetria/ Comentários
**Aparência facial** (em repouso e quando está chorando ou sendo estimulado)	Sorri ou reage a estímulos fechando os olhos e fazendo caretas		Fecha os olhos, mas não aperta, pobre expressão facial	Sem expressões, não reage à estímulos		
**Movimentos dos olhos**	Movimentos normais e coordenados (ambos os lados)		**Intermitente** Desvio dos olhos ou movimentos anormais	**Contínuo** Desvio dos olhos ou movimentos anormais		
**Reposta visual** Testar a capacidade de acompanhar um alvo preto/branco	Acompanha o alvo em um arco completo		Acompanha o alvo em um arco incompleto ou assimétrico	Não acompanha o alvo		
**Reposta auditiva** Testar a resposta a um chocalho	Reage a estímulos de ambos os lados		Reação duvidosa aos estímulos ou assimetria de resposta	Sem resposta		
**Sugar/Deglutir** Observe a criança sugando o seio materno ou mamadeira. Se for mais velha, pergunte sobre alimentação, tosse associada e recusa excessiva	Boa sucção e deglutição		Pobre sucção e/ou deglutição	Sem reflexo de sucção, sem deglutição		

Esc. = Escore

AVALIAÇÃO DA POSTURA (observe quaisquer assimetrias)

	Escore 3	Escore 2	Escore 1	Escore 0	Esc.	Assimetria/ Comentários
**Cabeça**<br>Sentado	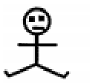<br>Reta; na linha média		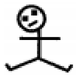 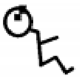<br>Levemente para o lado ou para trás ou para frente	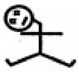 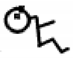<br>Acentuadamente para o lado ou para trás ou para frente		
**Tronco**<br>Sentado	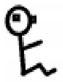<br>Reto		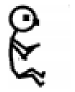<br>Levemente curvado ou inclinado para o lado	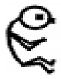 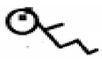 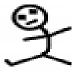<br>Muito Curvado / Inclinando para trás / Inclinado para o lado		
**Braços**<br>Em repouso	Em posição neutra, retos no centro ou levementefletidos		**Leve**<br>Rotação interna ou rotação externa<br><br>**Intermitente**<br>Postura distônica	**Acentuado**<br>Rotação interna ou rotação externa<br><br>Postura distônica<br>Postura hemiplégica		
**Mãos**	Mãos abertas		**Intermitente**<br>Polegar aduzido ou mão fechada	**Persistente**<br>Polegar aduzido ou mão fechada		
**Pernas**<br>Sentado<br><br>Supino e em pé	Capaz de sentar com o tronco reto e pernas retas ou levemente fletidas (permanece muito tempo sentado)<br>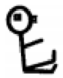<br>Pernas em posição neutra, retas ou levemente fletidas	**Leve**<br>Rotação interna ou rotação externa	Senta com o tronco reto mas com os joelhos fletidos a 15-20º<br>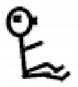<br>Rotação interna ou rotação externa dos quadris	Incapaz de sentar reto, a menos que os joelhos estejam acentuadamente fletidos (não permanece muito tempo sentado)<br>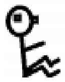<br>**Acentuada**<br>Rotação interna ou rotação externa ou extensão ou flexão fixada ou contraturas nos quadris e joelhos		
**Pés**<br>Em supino e em pé	Centralizados na posição neutra<br><br>Dedos retos em semi flexão ou extensão (em posição neutra)		**Leve**<br>Rotação interna e rotação externa<br><br>**Intermitente**<br>Tendência de ficar em pé na ponta dos pés ou com dedos fletidos ou estendidos	**Acentuada**<br>Rotação interna ou rotação externa do tornozelo<br><br>**Persistente**<br>Tendência de ficar em pé na ponta dos pés ou com dedos fletidos ou estendidos		

Esc. = Escore

AVALIAÇÃO DOS MOVIMENTOS

	Escore 3	Escore 2	Escore 1	Escore 0	Esc.	Assimetria/ Comentários
**Quantidade**<br>Observe a criança deitada em supino	Normal		Excessivo ou lento	Mínimo ou nenhum		
**Qualidade**<br>Observe a atividade motora espontânea voluntária do bebê durante a avaliação	Livre, alternado e suave		Brusco<br><br>Leve tremor	▪ Rígido e sincrônico<br>▪ Espasmos extensores<br>▪ Atetóide<br>▪ Atáxico<br>▪ Muito trêmulo<br>▪ Espasmo mioclônico<br>▪ Movimento distônico		

Esc. = Escore

AVALIAÇÃODO TÔNUS

	Escore 3	Escore 2	Escore 1	Escore 0	Esc.	Assimetria/ Comentários
**Sinal do cachecol**<br>Pegue a mão da criança e cruze o braço à frente do peito até encontrar resistência. Observe a posição do cotovelo em relação a linha média.	**Amplitude:**<br>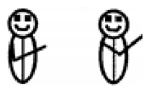<br>**D E D E**		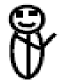<br>**D E**	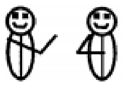<br>**D E ou D E**		
**Elevação passiva do ombro**<br>Levante o braço ao lado da cabeça da criança. Observe a resistência no ombro e cotovelo.	Resistência superável<br>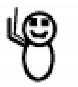<br>**D E**	Resistência difícil de superar<br>**D E**	Sem resistência<br>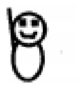<br>**DE**	Resistência, não superável<br>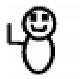<br>**DE**		
**Pronação/supinação**<br>Estabilizeo braço enquanto é feito a pronação e supinação no antebraço, observe a resistência	Pronação completa e supinação, sem resistência		Resistência total à pronação completa/ supinação superável	Não é possível pronação completa e supinação, resistência acentuada		
**Adutores de quadril**<br>Com as duas pernas da criança estendidas, faça abdução de ambos os membros, o mais distante possível. Observe o ângulo formado pelas pernas.	**Amplitude: 150-80º**<br>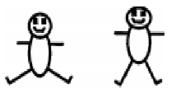<br>**D E D E**	**150-160º**<br>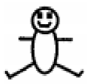<br>**D E**	**>170º**<br>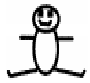<br>**D E**	**<80º**<br>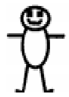<br>**D E**		
**Ângulo poplíteo**<br>Mantendo as nádegas da criança na cama, flexione os quadris sobre o abdômen, depois estenda os joelhos até encontrar resistência. Observe o ângulo entre a parte superior e inferior da perna.	**Amplitude: 150-100º**<br>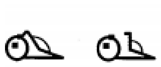<br>**D E D E**	**150-160º**<br>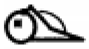<br>**D E**	**~90º ou >170º**<br>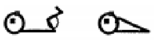<br>**D E D E**	**<80º**<br>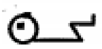<br>**D E**		
**Dorsiflexão de tornozelo**<br>Com o joelho estendido, faça dorsiflexão do tornozelo. Observe o ângulo entre o pé e a perna.	**Amplitude: 30º-85º**<br>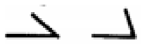<br>**D E D E**	**20-30º**<br>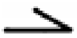<br>**D E**	**<20º ou 90º**<br>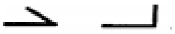<br>**D E D E**	**>90º**<br>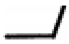<br>**D E**		
**Puxado para sentar**<br>Puxe a criança para sentar pelos punhos (se necessário, apoie a cabeça)	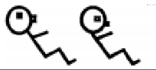		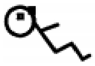	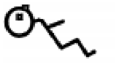		
**Suspensão ventral**<br>Segure a criança horizontalmente, em suspensão ventral, pelo tronco; observe a posição da coluna, membros e cabeça.	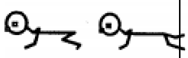		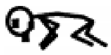	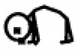		

Esc. = Escore

REFLEXOS E REAÇÕES

	Escore 3	Escore 2	Escore 1	Escore 0	Esc.	Assimetria/ Comentários
**Proteção do braço**<br>Puxe a criança por um braço na posição supina (estabilize o quadril contralateral) e observe a reação do braço no lado oposto.	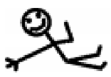<br>Braço e mão estendidos<br>**D E**		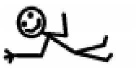<br>Braço semiflexionado<br>**D E**	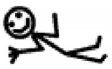<br>Braço completamente flexionado<br>**D E**		

**Suspensão vertical** Segure a criança embaixo das axilas, não deixando que as pernas toquem na superfície – você pode fazer "cócegas" nos pés para estimular os chutes.	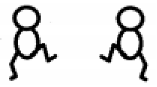 Chuta simetricamente		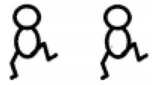 Chuta mais uma perna ou chutes fracos	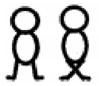 Não chuta mesmo que estimulado ou em posição de tesoura		
**Inclinação lateral** (descreva o lado que está para cima). Segure a criança verticalmente próximo dos quadris e incline lateralmente em direção horizontal. Observe a resposta do tronco, coluna, membros e cabeça.	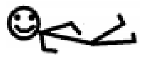	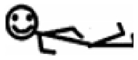	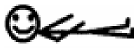	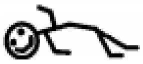		
**Paraquedas anterior** Segure a criança verticalmente e rapidamente incline-a para frente. Observe a reação/simetria da resposta dos braços.	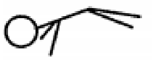 (após 6 meses)		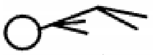 (após 6 meses)			
**Reflexos tendinosos** Com a criança relaxada, sentada ou deitada – use um martelo pequeno	Facilmente excitável<br><br>bíceps<br>joelho<br>tornozelo<br><br>(reflexos normais)	Levemente ativo<br><br>bíceps<br>joelho<br>tornozelo<br><br>(reflexos hipoativos)	Ativos<br><br>bíceps<br>joelho<br>tornozelo<br><br>(reflexos hiperativos)	Clônus ou ausente<br><br>bíceps<br>joelho<br>tornozelo		

Esc. = Escore

SEÇÃO 2:MARCOS MOTORES (não pontuados; observe assimetrias)

**Controle da cabeça**	Incapaz de manter a cabeça levantada normal aos 3 meses	Oscilações<br><br>normal até 4 meses	Mantém a cabeça ereta todo o tempo<br><br>normal a partir de 5 meses			Por favor, anotar idade em que a habilidade máxima é alcançada
**Sentar**	Não pode sentar	Com suporte nos quadris 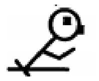 normal aos 4 meses	Escorado 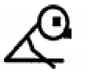 normal aos 6 meses	Senta-se estável 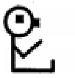 normal aos 7-8 meses	Pivoteia (gira) 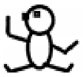 normal aos 9 meses	Observado:<br><br>Relatado (idade):
**Preensão voluntária – observe o lado**	Sem preensão	Usa toda mão	Dedo indicador e polegar, mas preensão imatura	Preensão em pinça		Observado:<br><br>Relatado (idade):
**Habilidade de chutar em supino**	Não chuta	Chuta horizontalmente, mas não eleva as pernas	Para cima (verticalmente) 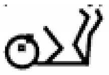 normal aos 3 meses	Toca a perna 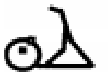 normal aos 4-5 meses	Toca nos dedos dos pés 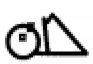 normal aos 5-6 meses	Observado:<br><br>Relatado (idade):
**Rolar – observe para qual lado**	Não rola	Rola para o lado normal aos 4 meses	Rola de prono para supino normal aos 6 meses	Rola de supino para prono normal aos 6 meses		Observado:<br><br>Relatado (idade):
**Arrastar/engatinhar – observa se arrasta sentado**	Não levanta a cabeça	Com cotovelo	Com mãos estendidas	Rasteja/engatinha com abdômen	Engatinha com mãos e joelhos	Observado:<br><br>Relatado (idade):

		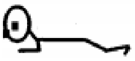 normal aos 3 meses	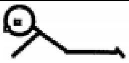 normal aos 4 meses	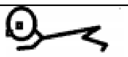 normal aos 8 meses	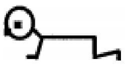 normal aos 10 meses	
**Em pé**	Não sustenta o peso	Sustenta o peso<br><br>normal aos 4 meses	Em pé com apoio<br><br>normal aos 7 meses	Em pé sem ajuda<br><br>normal aos 12 meses		Observado:<br><br>Relatado (idade):
**Marcha**		Saltando<br><br>normal aos 6 meses	Cruzando (se segurando)<br><br>normal aos 12 meses	Marcha independente<br><br>normal aos 15 meses		Observado:<br><br>Relatado (idade):

SEÇÃO 3: COMPORTAMENTO (não pontuado)

	1	2	3	4	5	6	Comentário
**Estado consciente**	Irritado	Sonolento	Dormindo, mas acorda facilmente	Acordado, mas sem interesse	Perde interesse	Mantém interesse	
**Estado emocional**	Irritável, não consolável	Irritável, cuidador consola	Irritável quando se aproxima	Nem feliz ou infeliz	Feliz e sorridente		
**Orientação social**	Evitando, retirando	Hesitante	Aceita aproximação	Amigável			

* Tradução da versão brasileira (2020), Michelle Alexandrina dos Santos Furtado (alexandrinamichelle@gmail.com) UFVJM/MG, Hércules Ribeiro Leite (hercules@ufmg.br) UFMG/MG, Tathiana Ghisi de Souza (tathi_neo@yahoo.com.br), Kênnea Martins Almeida (kennea.almeida@gmail.com) UnB/DF, Ana Cristina Resende Camargos (anacristinarcamargos@gmail.com) UFMG/MG, Ayrles Silva Gonçalves Barbosa Mendonça (ayrles @ yahoo .com.br) UFAM/AM, Matheus Rocha Pereira Klettenberg (mklettenberg@gmail.com) UnB/DF e Victor Alves Rodrigues (victoralvesrodrigues2017@gmail.com) UnB/DF.

Este é o formulário oficial para o exame neurológico infantil Hammersmith.
Seu conteúdo e sistema de pontuação não devem ser alterados. Referência principal Haataja L et al J Peds 1999; 135: 153-61
Para perguntas sobre o exame, entre em contato com Prof Frances Cowan f.cowan@imperial.ac.uk,
Prof. Leena Haataja leena.haataja@hus.fi ou Prof Eugenio Mercuri eugeniomercuri@unicatt.it
Site hammersmith-neuro-exam.com
